# Thrombin Generation in Acute Ischaemic Stroke

**DOI:** 10.1155/2016/7940680

**Published:** 2016-12-25

**Authors:** Ibrahim O. Balogun, Lara N. Roberts, Raj Patel, Rohan Pathansali, Lalit Kalra, Roopen Arya

**Affiliations:** ^1^King's Thrombosis Centre, Department of Haematological Medicine, King's College Hospital NHS Foundation Trust, London, UK; ^2^Department of Stroke Medicine, East Kent Hospitals University NHS Foundation Trust, East Kent, UK; ^3^Department of Stroke Medicine, King's College Hospital NHS Foundation Trust, London, UK; ^4^Clinical Neuroscience Department, Academic Neuroscience Centre, King's College London, London, UK

## Abstract

*Introduction*. Stroke remains a global leading cause of death and disability. Traditional description of plasma biology in the aftermath of acute ischaemic stroke favours development of hypercoagulability, resulting from complex interplay between plasma and endothelial factors. However, no single assay measures the overall global coagulation process. We postulate that thrombin generation would assist in identifying coagulation abnormalities after acute stroke.* Aim*. To investigate the coagulation abnormalities after acute ischaemic stroke using thrombin generation.* Methods*. We evaluated thrombin generation, measured with calibrated automated thrombography in stroke of different aetiological types (*n* = 170) within 48 hours of symptoms onset (baseline) and in the second week (time 2) and in normal healthy volunteers (*n* = 71).* Results*. Two-point thrombin generation assays showed prolonged lag time and time to peak at baseline (3.3 (2.9, 4.0) versus 3.6 (3.2, 4.7); *p* = 0.005) and (3.3 (2.9, 4.0) versus 3.6 (3.2, 4.7); *p* = 0.002), respectively, and at time 2 (3.5 (2.9, 4.2) versus 4.0 (3.1, 4.9); *p* = 0.004) and (5.9 (5.3, 6.6) versus 6.8 (5.8, 7.7) *p* = 0.05), respectively, in cardioembolic stroke (*n* = 39), when compared to noncardioembolic stroke (*n* = 117). The result was reproduced in multiple comparisons between acute ischaemic stroke subgroups and normal healthy volunteers. Endogenous thrombin potential and peak thrombin did not indicate hypercoagulability after acute ischaemic stroke, and thrombolytic therapy did not affect thrombin generation assays.* Conclusion*. Our findings suggest that thrombin generation in platelet poor plasma is not useful in defining hypercoagulability in acute ischaemic stroke. This is similar to observed trend in coronary artery disease and contrary to other hypercoagulable states.

## 1. Background

Stroke is a global leading cause of death and disability [1, 2]. Despite improvement in therapeutic options, the mechanism underpinning development of a hypercoagulable state in the aftermath of acute ischaemic stroke remains unclear. Plasma and endothelial factors play significant roles in determining damage and repair of vascular injuries [3–5], and development of hypercoagulability after acute stroke is dependent upon a complex interaction between plasma and endothelial factors which can be measured in vitro [3, 6, 7]. However, wide observer variability, limited biological assay techniques, and lack of specificity precluded routine measurement and clinical applications [3, 6–9]. Thrombin, which is a protease enzyme, plays a central role in this interaction [10–13]. The focus on thrombin generation is essential and timely, considering peripheral assay is a test of global in vivo plasma thrombin generation potential, when compared to the traditional clotting time and in comparison to other nonspecific plasma and endothelial markers [11, 12, 14]. Such a role ascribed to thrombin has been examined, with elevated endogenous thrombin potential (ETP) and peak thrombin levels demonstrated in venous thromboembolism and acute coronary artery disease and speculated in ischaemic stroke [12, 15–17]. There is increasing speculation about dissimilar trend in thrombin generation kinetics in VTE which showed elevated ETP and PTG, when compared to coronary artery disease with elevated lag time and time to peak [15, 18–20]. Such discordant trend has been suggested in stroke of different aetiological types [16, 21]. Modern understanding of pathogenesis of lacunar stroke and large artery atherosclerosis suggests a common underlying aetiology, and as such they are increasingly grouped as noncardioembolic stroke [22–24]. We postulate that thrombin generation assays would identify hypercoagulable states in the aftermath of acute ischaemic stroke, which could assist in further understanding the disease modifying roles of direct thrombin inhibitors in secondary prevention of ischaemic brain damage.

## 2. Study Objectives

1. To evaluate thrombin generation in acute stroke.
2. To establish difference in thrombin generation between stroke subtypes and control group.
3. To examine the effect of thrombolytic therapy on thrombin generation.

## 3. Materials and Methods

### 3.1. Study Design

Participant recruitment into subject group was from among prospective in-patient stroke admissions at King's College Hospital NHS Foundation Trust, between June 2009 and August 2011. Normal healthy volunteers without cardiovascular and cerebrovascular diseases risk factors were invited from relatives, friends, and carers of participants in the subject group. All participants in this study were from the original study which examined clinical and laboratory predictors of DVT after acute stroke [25]. Following objective diagnosis of stroke, participants were invited to take part within 48 hours of symptom onset. Excluded are those with early repatriation to local hospitals, recent or previous diagnosis of VTE, anticoagulation, previous diagnosis of cancer, radiological diagnosis of stroke after 48 hours of symptoms onset, and lower limb amputation. Clinicodemographic data obtained were National Institute of Health Stroke (NIHSS), Barthel Index (BI), height (m), weight (kg), past medical history, and time to acquisition of tested variables. Radiological diagnosis of large artery atherosclerosis and lacunar stroke was based on computerised tomography (CT) and computerised tomography angiogram (CTA) and magnetic resonance imaging (MRI) and contrast enhanced magnetic resonance angiogram (CEMRA) studies, carried out within 24 hours of when a subject last felt or was seen normal. Diagnosis of cardioembolic stroke was based on routine admission 12-lead ECG or subsequent 24-hour ECG confirming atrial fibrillation. Ischaemic stroke was classified according to Trial of ORG 10172 in acute stroke treatment (TOAST) criteria [26]. Included in the study are participants with cardioembolic and noncardioembolic (large artery atherosclerosis and lacunar) stroke. Patients with noncardioembolic stroke received Aspirin and Dipyridamole or Clopidogrel after 24 hours of admission, while cardioembolic stroke received Aspirin initially and then later commenced formal anticoagulation with warfarin or heparin ≥2 weeks after diagnosis. Patients with stroke of undetermined aetiology and two or more causes were excluded, so as to avoid the effect of multiple aetiology (or no aetiology) on the final data analysis. The study was approved by York Research Ethics Committee (REC reference number: 09/H1311/12) and the local Research and Development department at King's College Hospital, London. Informed written consent and when applicable assent were obtained prior to data and sample collection.

### 3.2. Blood Samples

#### 3.2.1. Sample Collection and Preparation

Venous blood sampling was carried out within 48 hours from symptom onset. Venepuncture was from the antecubital veins, with minimal venous stasis using a Butterfly-21 needle (Hospira Inc, IL, USA). The first 10 mL collected was discarded, and the second 10 mL was divided into eight 0.109 M trisodium citrate BD vacutainers (BD Diagnostics, Plymouth, UK). The first 10 mL of blood is divided into two 0.369 M ethylenediamine tetraacetic acid (EDTA) BD vacutainer (BD Diagnostics, Plymouth, UK) and two serum SST BD vacutainer with clot activator (BD Diagnostics, Plymouth, UK). All samples were transported at room temperature prior to processing.

Preparation of platelet poor plasma (PPP) for thrombin generation assay was by double centrifugation (Hettich Rotina 46R Rotina Centrifuge, Tuttlingen, Germany) at 4750*g* for 10 minutes, at room temperature. After the initial centrifugation, the top three-quarters of supernatant was pipetted into a polypropylene tube prior to second centrifugation. The top three-quarter supernatant was then removed and stored at −40°C. Samples were processed and frozen within 30 minutes of collection.

Plasma samples for D-dimer, Clauss fibrinogen, were prepared by single centrifugation at 3040*g* for 7 minutes, at room temperature. Thrombin generation was performed within 12 weeks of sample collection. PPP was then thawed in a water bath at 37°C and centrifuged as above immediately prior thrombin generation testing.

### 3.3. Laboratory Assays

#### 3.3.1. Thrombin Generation

Thrombin generation was measured in PPP with calibrated automated thrombography (Thrombinoscope BV, Maastrichts, Netherlands) as previously detailed [27, 28]. All samples were tested in duplicate. Final tissue factor concentration was 5 pM with 4 *μ*m of phospholipids. Lag time (minutes), time to peak (minutes), peak thrombin (nM), and endogenous thrombin potential (ETP), which is the area under the curve (nM·min), were the parameters generated.

Intra- and interassay variability were <5% and <12%, respectively, for all parameters.

### 3.4. Standard Coagulation Assays

D-dimer was measured by latex photometric immunoassay and fibrinogen was by Clauss method using Diagnostica Stago reagents (Asnieres, France) on the automated STA® Evolution analyser (Diagnostica Stago) as previously described [29].

### 3.5. Statistical Analysis

#### 3.5.1. Sample Size

We have previously demonstrated significant difference in thrombin generation in an ethnographic based study, with a small sample size of 26 participants per group which provided 80% power to detect significant difference at 5% confidence level [28]. Peak thrombin generation was significantly higher in African-Caribbeans with DVT, 399.7 ± 53.6, and in African-Caribbean controls, 356.1 ± 53.7.

Normally and nonnormally distributed variables between ischaemic stroke subgroups and those with recombinant tissue plasminogen activator (rtPA) were analysed using Fisher's exact test, unpaired* t*-test, and Mann–Whitney* U* test. Logistic regression model was used in the comparison between ischaemic stroke subgroups and in patients treated with (rtPA), with simultaneous adjustment for age, male gender, baseline stroke severity, hypertension, ischaemic heart disease (IHD), atrial fibrillation (AF), and African-Caribbean ethnicity. Corresponding odds ratio and confidence interval were derived from the regression analysis. Missing data was handled by single imputation method, which replaces missing values with predicted scores from a regression equation, and this is based on information from the observed data. Further analysis compared thrombin generation and other markers of haemostatic activation between each stroke subtype and the control group. This analysis was performed by splitting data accrued into four groups. These were control, noncardioembolic, cardioembolic, and haemorrhagic groups. ANOVA by ranks was used in the multiple comparisons. And, in case of a significant omnibus test, a further post hoc adjustment to *p* value was according to Bonferroni procedure. Statistical significance was given a *p* value of <0.05. All statistical analyses were performed using Stata version 12 software (Stata Corp LP, Texas, USA).

## 4. Results

### 4.1. Participants Characteristics

**Figure 1:**
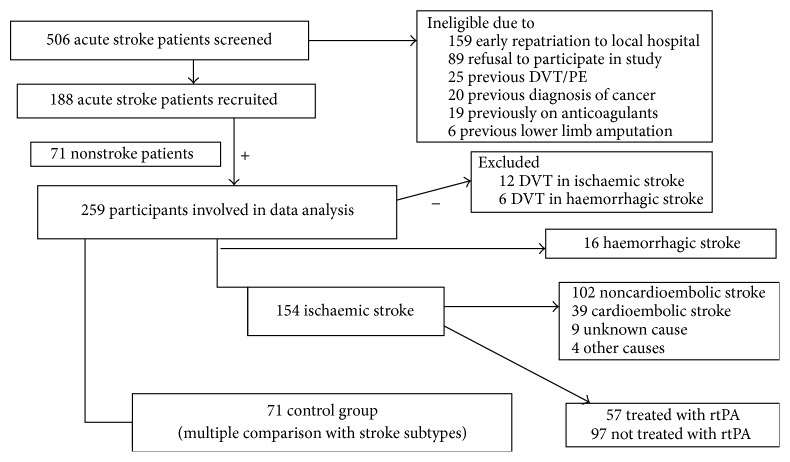
Flowchart of recruitment of participants. AIS: acute ischaemic stroke; DVT: deep vein thrombosis; PE: pulmonary embolism; rtPA: recombinant tissue plasminogen activator.

Five hundred and six participants were screened, with exclusions as shown in Figure 1. 241 participants were recruited, from which 188 were in the subject and 71 in the control group. 57 subjects received thrombolytic therapy. 18 subjects had asymptomatic DVT and were excluded. In Table 1 is a summary of participant demographic details in both the subject and control group. There were 59.5% noncardioembolic stroke (lacunar (39%), large artery atherosclerosis (20.5%)) and 22.9% cardioembolic stroke. Median time (IQR) to sample collection was 19 hours [10, 30].

### 4.2. Subgroup Characteristics

In Table 2, noncardioembolic subjects were predominantly hypertensive, while cardioembolic stroke had more subjects with coronary artery disease and dyslipidaemia.

### 4.3. Thrombin Generation in Cardioembolic versus Noncardioembolic Stroke

The results in Table 3 showed thrombin generation in cardioembolic stroke, detailing prolonged lag time (*p* = 0.005) and ttP (*p* = 0.002) at base line and time 2 and lag time (*p* = 0.004) and ttP (*p* = 0.05) with corresponding elevated D-dimer (*p* = 0.0001) at baseline and time 2. There was no significant difference between thrombin generation parameters, D-dimer, or fibrinogen measured at time 1 and time 2.

### 4.4. Thrombin Generation in Stroke Subtypes and Healthy Controls

Table 4 shows baseline thrombin generation, D-dimer, and fibrinogen in stroke subtypes compared to controls. Lag time (*p* < 0.001) and ttP (*p* < 0.001) were significantly prolonged in cardioembolic stroke, when compared to other stroke types and normal healthy volunteers, with significantly elevated D-dimer (*p* < 0.001).

### 4.5. Thrombin Generation in Patients Treated with rtPA

The thrombin generation parameters were not significantly different between the two groups; refer to Table 5. There was significantly elevated fibrinogen at baseline (*p* = 0.006) in patients treated with rtPA. There was no significant difference between thrombin generation parameters, D-dimer, or fibrinogen measured at time 1 and time 2.

## 5. Discussion

Elevated lag time and time to peak in the aftermath of acute stroke in our study population suggest that thrombin generation measured by CAT is not a sensitive measure to hypercoagulability associated with acute stroke. This is in keeping with inverse relationship observed in other studies following acute myocardial infarction (AMI) and stroke [15, 31]. Conversely, in another study, thrombin generation following acute stroke measured within 2 weeks and at 1 month after stroke showed elevated ETP and peak thrombin concentrations [16, 32], similar to other hypercoagulable states such as VTE and age in hormone replacement therapy [18, 20, 29, 33]. Our findings may differ due to earlier initial measurement of thrombin generation, inclusion of participants with AF at time of recruitment, and differences in demographic profiles and stroke severity between the two populations examined. Our observation may create further inroad into understanding difference in thrombin generation between VTE and arterial disease such as acute stroke. Recently, a common pathogenic process has been ascribed to development of atherosclerotic and arteriolosclerotic diseases in coronary artery disease, lacuna stroke, and large artery atherosclerosis [22]. The role of thrombin in this pathogenic process has not been previously explained. This common pathogenic pathway may provide useful explanation for elevated lag time and time to peak and lack of difference in plasma peak thrombin and ETP observed in our patient population and in AMI [15].

Elevated lag time and ttP observed in our study population may be explained by “*paradoxical or compensatory coagulation theory*” which we propose involves downregulation of initiation of the coagulation cascades from plasma factors which are normally switched on to avoid excessive hypercoagulability under physiologic conditions. In sustained plasma hypercoagulability due to atherothrombotic and cardioembolic process in micro- and macrovasculature, a feedback mechanism exists, which then favours anticoagulation rather than procoagulation, a process similar to physiologic haemostatic activation [12]. This process of microvascular thrombosis, subsequent endothelial perturbation, and eventual thrombin generation is mediated by plasma factors, such as tissue factor (TF), tissue factor pathway inhibitor (TFPI), thrombin activable fibrinolysis inhibitor (TAFI), and activated factor VIII (FVIIa) [12, 15]. TFPI has been shown to be a significant determinant of lag time and negatively correlates with ETP and peak thrombin in both healthy controls and AMI patients [15]. This effect is only seen in vitro and it is not likely to have an in vivo effect.

Smid et al. theorised an in vitro “anticoagulant” role for D-dimer in explaining prolongation of lag time observed in acute coronary disease, with elevated levels attributed to acute and sustained vascular injury [15]. Elevated D-dimer was observed in our patient population with concurrently elevated lag time levels, similar to observation in AMI. It is possible that TFPI may explain the lack of significant difference in ETP and peak thrombin concentration observed in our study. Considering we initially set out to evaluate global coagulation profile after acute ischaemic stroke, therefore we did not measure TFPI or other individual coagulation factors and cannot confirm this.

The lack of difference in thrombin generation between rtPA and nontreated groups was unexpected. Previous studies showed paradoxical rise in markers of in vivo thrombin generation after rtPA therapy [34, 35]. To our knowledge there are no studies of in vitro thrombin generation following rtPA treatment. We reproduced low level of plasma fibrinogen in rtPA therapy, which is due to blockade of formation of fibrin degradation products; this reflects similar actions from the naturally occurring tPA [36].

## Figures and Tables

**Table 1:** Demographic characteristics of all participants.

Criteria	Subject (*n* = 170)	Control (*n* = 71)
Age, mean (SD)	71 ± 11.4	70 ± 17.6
Male, *n* (%)	44 (23.4)	26 (37.0)
Ethnicity		
African-Caribbean, *n* (%)	58 (30.9)	26 (36.6)
Caucasians, *n* (%)	130 (69.1)	45 (63.3)
Risk factors		
Hypertension, *n* (%)	111 (59.0)	7 (9.9)
Diabetes mellitus, *n* (%)	43 (22.3)	3 (4.2)
Smoking, *n* (%)	31 (16.5)	2 (2.2)
Atrial fibrillation, *n* (%)	43 (22.9)	—
Dyslipidaemia, *n* (%)	47 (25.0)	—
IHD, *n* (%)	43 (22.9)	—
Peripheral vascular disease, *n* (%)	6 (3.2)	—
BMI, mean kg/m^2^ (SD)	27.6 ± 5.7	28 ± 5.6
Medications		
Antiplatelets, *n* (%)	14 (7.5)	—
Statins, *n* (%)	44 (23.4)	—
Antihypertensive, *n* (%)	59 (31.4)	5 (7)
rtPA, *n* (%)	63 (33.5)	(—)
No rtPA, *n* (%)	109 (58.0)	(—)
Stroke type (TOAST criteria)		
ICH, *n* (%)	16 (9.4)	(—)
Large artery atherosclerosis, *n* (%)	35 (20.5)	(—)
Lacunar, *n* (%)	67 (39.4)	(—)
Cardioembolic, *n* (%)	39 (22.9)	(—)
Unknown, *n* (%)	9 (5.2)	(—)
Other causes, *n* (%)	4 (2.3)	(—)
Time to acquisition of tested variables		
NIHSS, median (IQR), min	28 (17, 42)	(—)
Barthel index, median (IQR), min	28 (17, 42)	(—)
NCCT, median (IQR), hours	3.8 (0.5, 23)	(—)
Sample collection, median (IQR), hours	19 (10, 36)	(—)
DtN time (rtPA), median (IQR), hours	47 (34, 84)	(—)

(—): not relevant; IHD: ischaemic heart disease; rtPA: recombinant tissue plasminogen activator; PICH: primary intracerebral haemorrhage; NIHSS: National Institute of Health Stroke scale; NCCT: noncontrast computerised tomography; DtN: door to needle time.

**Table 2:** Baseline characteristics in ischaemic stroke subgroup.

Category	Noncardioembolic (*n* = 102)	Cardioembolic (*n* = 39)	*p* value
Age, mean (SD)	70 ± 14	74 ± 13	0.75
Male, *n* (%)	73 (57.5)	18 (46.2)	0.84
Ethnicity			
Caucasians, *n* (%)	80 (78.4)	29 (74.4)	0.18
African-Caribbean, *n* (%)	32 (31.4)	10 (25.6)	0.20
Risk factors			
Diabetes mellitus, *n* (%)	27 (31.3)	6 (15.4)	0.06
Hypertension, *n* (%)	66 (64.7)	19 (48.7)	**0.006**
Smoking, *n* (%)	20 (19.6)	6 (15.4)	0.20
Dyslipidaemia, *n* (%)	23 (22.5)	16 (41.0)	**0.004**
Ischaemic heart disease, *n* (%)	23 (22.5)	13 (33.3)	**0.02**
Peripheral vascular disease, *n* (%)	3 (2.9)	1 (2.5)	0.4
Prestroke medications			
Antiplatelets, *n* (%)	5 (4.9)	2 (5.1)	0.31
Statins, *n* (%)	19 (18.6)	10 (25.6)	0.16
Antihypertensives, *n* (%)	17 (16.7)	29 (74.4)	0.06
rtPA, *n* (%)	35 (34.3)	12 (30.8)	0.23
Body mass Index (kg/m^2^)			
BMI-mean (SD), *n* (%)	26.7 ± 3.8	28.6 ± 3.4	0.63
Stroke severity			
NIHSS- mean (SD)	8.0 ± 7.1	15.6 ± 7.7	0.07

**Table 3:** Thrombin generation in ischaemic stroke subtypes.

Category	Noncardioembolic time 1 (*n* = 102) time 2 (80)	Cardioembolic time 1 (*n* = 39) time 2 (*n* = 37)	*p* value
*Thrombin generation assays*			
Median lag time 1, min (IQR)	3.3 (2.9, 4.0)	3.6 (3.2, 4.7)	**0.005**
Median lag time 2, min (IQR)	3.5 (2.9, 4.2)	4.0 (3.1, 4.9)	**0.004**
Median difference, 95% CI	−0.20 (−0.61–0.21)	−0.40 (−1.06–0.26)	
Mean ETP 1, nm min (SD)	1766 ± 385	1816 ± 357	0.50
Mean ETP 2, nm min (SD)	1715 ± 349	1696 ± 230	0.81
Mean difference, 95% CI	51.00 (−55.10–157.0)	120 (−18.08–258.08)	
Median ttP 1, min (IQR)	3.3 (2.9, 4.0)	3.6 (3.2, 4.7)	**0.002**
Median ttP 2, min (IQR)	5.9 (5.3, 6.6)	6.8 (5.8, 7.7)	**0.05**
Median difference, 95% CI	−0.20 (−0.60–0.21)	−0.20 (−0.86–0.46)	
Mean peak 1, nm (SD)	335 ± 72	346 ± 71	0.94
Mean peak 2, nm (SD)	348 ± 77	345 ± 63	0.87
Mean difference, 95% CI	−13.0 (−34.19–8.19)	1.00 (−29.74–31.74)	
*Standard haemostatic markers*			
Median D-dimer 1, ng/mL (IQR)	1215 (590, 2670)	1480 (770, 3780)	**0.0001**
Median D-dimer 2, ng/mL (IQR)	1120 (480, 2960)	1275 (720, 2210)	**0.0001**
Median difference, 95% CI	95 (−61.82–251.82)	205 (−136.17–546.17)	
Mean fibrinogen 1, g/L (SD)	3.9 ± 1.4	3.7 ± 1.3	0.66
Mean fibrinogen 2, g/L (SD)	4.5 ± 1.3	4.6 ± 1.3	0.90
Mean difference, 95% CI	−0.60 (−0.99–−0.21)	−0.90 (−1.49–−0.31)	

**Table 4:** Thrombin generation in acute stroke versus control.

Parameters	Group (*n* = 228)	Summary	*p* value versus control^(†)^
Median Lag time, min (IQR)	Control	2.85 (2.56, 3.33)	
	Noncardioembolic	3.25 (2.91, 4.00)	**<0.001**
	Cardioembolic	3.63 (3.22, 4.67)	**<0.001**
	PICH	3.22 (3.00, 4.14)	0.06

Mean ETP, nM min (SD)	Control	1737 ± 326	
	Noncardioembolic	1766 ± 385	1.00
	Cardioembolic	1816 ± 357	0.96
	PICH	1852 ± 424	0.77

Median ttP, min (IQR)	Control	5.52 (5.00, 6.25)	
	Noncardioembolic	5.67 (5.21, 6.33)	0.72
	Cardioembolic	6.58 (5.67, 7.56)	**<0.001**
	PICH	6.00 (5.40, 6.84)	0.43

Median peak, nM (SD)	Control	331 ± 58	
	Noncardioembolic	345 ± 72	0.59
	Cardioembolic	346 ± 71	0.90
	PICH	330 ± 78	1.00

Median D-dimer, ng/mL (IQR)	Control	450 (300, 1070)	
	Noncardioembolic	1215 (590, 2670)	**<0.001**
	Cardioembolic	1480 (770, 3780)	**<0.001**
	PICH	1040 (530, 1470)	0.14

Mean fibrinogen, g/L (SD)	Control	3.82 ± 1.11	
	Noncardioembolic	3.86 ± 1.37	1.00
	Cardioembolic	3.74 ± 1.32	1.00
	PICH	3.94 ± 1.32	1.00

^(†)^ *p* values given Bonferroni adjustment to allow for multiple comparisons. Control (*n* = 71), noncardioembolic (*n* = 102). Cardioembolic (*n* = 39), PICH (*n* = 16).

**Table 5:** Haemostatic activation after rtPA therapy.

Category	No rtPA time 1 (*n* = 97) time 2 (*n* = 80)	rtPA time 1 (*n* = 57) time 2 (*n* = 54)	*p* value
*Thrombin generation assays*			
Lag time 1 (min)-median (IQR)	3.3 (3.0, 4.1)	3.3 (3.0, 4.0)	0.60
Lag time 2 (min)-median (IQR)	3.6 (2.9, 4.3)	3.3 (3.2, 4.0)	0.30
Median difference, 95% CI	0.30 (−0.70–0.10)	0.00 (−0.51–0.51)	
ETP 1 (nm min)-mean (SD)	1762 ± 342	1820 ± 449	0.37
ETP 2 (nm min)-mean (SD)	1714 ± 310	1683 ± 337	0.65
Mean difference, 95% CI	48.0 (−49.75–145.75)	137 (−12.98–286.98)	
ttP 1 (min)- median (IQR)	5.9 (5.3, 6.8)	6.0 (5.2, 6.9)	0.68
ttP2 (min)-median (IQR)	6.2 (5.6, 7.2)	5.9 (5.2, 6.6)	0.20
Median difference, 95% CI	−0.30 (−0.70–0.10)	0.10 (−0.41–0.61)	
Peak 1 (nm)-mean (SD)	346 ± 69	340 ± 78	0.64
Peak 2 (nm)-mean (SD)	345 ± 79	345 ± 60	0.99
Mean difference, 95% CI	1.00 (−20.96–22.96)	−5.00 (−31.28–21.28)	
*Standard coagulation assays*			
D-dimer 1 (ng/mL)-median (IQR)	1155 (580, 2145)	1670 (650, 3890)	0.07
D-dimer 2 (ng/mL)-median (IQR)	1100 (650, 2770)	1415 (480, 3050)	0.98
Median difference, 95% CI	55.0 (−127.59–237.59)	255.0 (39.08–470.92)	
Fibrinogen 1 (g/L)- mean (SD)	4.1 ± 1.3	3.4 ± 1.4	**0.006**
Fibrinogen 2 (g/L)-mean (SD)	4.7 ± 1.3	4.3 ± 1.3	0.08
Mean difference, 95% CI	−0.60 (−0.99–−0.21)	−0.9 (−1.41–−0.39)	

## Additional Points

The novel findings are as follows. (i) Thrombin generation profile is different in arterial disease. (ii) Lag time and ttP were prolonged in ischaemic stroke. (iii) Lag time and ttP in cardioembolic stroke are elevated following acute presentation and at week 2 when compared to noncardioembolic stroke. (iv) ETP and peak thrombin concentrations were not different between the stroke types or compared to healthy controls. And what we already know and we confirmed is as follows. (i) Fibrinogen concentration is suppressed following thrombolytic therapy. (ii) D-dimer is elevated in cardioembolic stroke.

*Study Strengths and Limitations*. Early recruitment of participants within 24–48 hours of admission allows for early assay of markers of haemostatic activation, and this presumably represents plasma changes in the immediate period after acute stroke. This mirrors the early recruitment methods in the acute coronary artery disease [15], and this approach allows for determining the mean or median difference between the two-point assays. Also, inclusion of participants treated with rtPA in the analysis provided new information on use of calibrated automated thrombography compared to previous studies where conclusions were drawn from plasma thrombin-antithrombin complex. Due to strict exclusion criteria, we are unable to offer detailed analysis in patients with stroke of yet undefined cause, and those with 2 or more causes. This is an important subject for future investigations, particularly in cryptogenic stroke. We recognise the effect of tissue factor concentrations on in vitro thrombin generation. It was measured at different concentrations (1 pM and 5 pM) in the coronary artery disease study [15]. Using a lower concentration of tissue factor would have necessitated use of CTI prior to sample collection [30]. Nonetheless, the use of a higher tissue concentration avoids this less cost-effective step and thus creates an opportunity for reproducibility of the technique [28]. In conclusion, we have demonstrated that thrombin generation is not sensitive to hypercoagulability in acute ischaemic stroke compared to controls. On the contrary, D-dimer is a better marker of hypercoagulability after acute ischaemic stroke.
